# Regional diversification of the use of agricultural production potential in the EU candidate and the Eastern partnership countries

**DOI:** 10.1371/journal.pone.0257490

**Published:** 2021-10-14

**Authors:** Anna Jankowska

**Affiliations:** Department of Economics and Economic Policy in Agribusiness, Faculty of Economics, Poznan University of Life Sciences, Poznan, Poland; Szechenyi Istvan University: Szechenyi Istvan Egyetem, HUNGARY

## Abstract

Diversification of the agricultural production potential often implies the differentiation of the achieved farming productivity due to its effect on the agricultural resources and structural processes. The article aims to examine the diversity of the production potential in the agricultural sectors of the EU candidate countries (CC) and the Eastern Partnership countries (EPC) and its impact on the variety of the achieved productivity, as well as to present changes in the analyzed indicators in the years 2006–2017. A synthetic measure of agricultural development and a linear regression analysis were applied in the article. The research revealed that Belarus may be distinguished with regard to its production potential, as well as the achieved productivity. In most countries (with the exception of Montenegro and Macedonia), an increase in the value of the synthetic measure of the possessed potential has been recorded in the studied period. However, the synthetic measure of the agricultural productivity level displayed an insignificant raise only in half of the countries surveyed.

## Introduction

One of the central elements of the development of each country’s economy is efficient and modern agriculture. Development of agriculture and rural areas should be understood as an increase in the value of social income, reflected in the rise in consumption rate and quality of life, as well as changes in the broadly understood social and economic structure of non-urbanized areas [1, 2].

A relevant element in the development of agriculture comprises of endogenous factors which determine the economic and social structure from within and result from the natural market processes. The most fundamental are the resources of basic production factors, i.e. land, fixed and financial capital, natural resources and human capital, the internal structure of agribusiness, i.e. the share of individual aggregates in the value of global production or in the total number of employees, the level of technical and economic efficiency of individual entities, expressing the efficiency of resource involvement, the quantity and quality of elements of the technical and social infrastructure of rural areas, the quality of social capital shaping institutional and interpersonal relations in the management processes, individual entrepreneurship and risk propensity among rural residents [1, 3–6]. The said elements crucially influence the development rate of the individual countries’ agricultural sectors [7], as well as enable to assess the diversity of agricultural potential and productivity between them.

The transition to a higher level of economic development is accompanied by the reduction of differences between the share of agriculture in the creation of GDP and employment. Regardless of the increase in agricultural productivity and production efficiency, a process of diminishing of agriculture as a manufacturing department in the national economy can be observed. However, the limits of this phenomenon, which can assume a dangerous shape of marginalization, are important. In the interest of both farmers and consumers is not to allow agriculture to be marginalized, as food provides for the basic needs of all people [8].

The works of Muravsky [9], Epshtein [10], McConnell and Bru [11], Svobodin [12], Vinnechek [13], Forteza et al. [14], are dedicated to the analysis of the concept of “productive potential and productivity”. Summarizing various works that have been undertaken on this subject, it should be noted that the production potential at the macro level is defined as “the regional volume of output that is possible to produce with the full use of available resources” [15].

In other words, production potential can simply be defined as an opportunity to produce a certain volume of output at a certain time. According to Forteza et al. [14], the productive potential is a complex socio-economic category, including the resource potential, the production process and the final product, which also implies a level of socio-economic effectiveness. This means that the production potential of an economy is the ability of the economy to use all its available resources, taking into account the maximum degree of efficiency of their use.

To implement economic activities, achieve the planned goals and solve the assigned tasks, the country must have necessary resources at its disposal. The key to a buoyant and boisterous economy is the availability of the necessary amount of resources, taking into account their rational use. Characterizing the resources that are at the country’s disposal, such concepts as “resource base” and “resource potential” are very often used. At present, there is no single approach to the definition of these concepts, and, in general, scientists understand under these terms the totality of all the resources that economic entities possess [15].

The production potential of agriculture as well as respective agricultural entities, i.e. farms, is a sum of natural resources, methods of their utilization, natural conditions, workforce, technical means and fundamental economic conditions [16, 17]. Barthelemy and David [18], as well as Pawlewicz and Pawlewicz [19], emphasized that the proof of the production potential and production capacity of agriculture is the presence of production resources. In turn, the ability to utilize the potential and develop optimum relations between production factors has an influence on the efficiency of the production process and work efficiency [15].

The size, quality and structure of production resources and their efficient use, apart from the social and economic system and economic policy, are the key factors determining the competitiveness of the specific economy and its sectors [19–21]. The factors shaping production potential efficiency include the directions of agricultural production, the intensity of management, relations between prices of respective factors and their actual availability [22]. Knowledge on the potential production capacity of agriculture is essential since it makes it possible to determine the directions of the agricultural sector development for a specific country or region.

So far, the research on agricultural sector’s development level has mainly focused on some aspects of the production potential and productivity in one country [23–43] or comperative analises of only a few countries studied by the authors, e.g. Burkitbayeva et al. [44] studied Albania, Armenia, Azerbaijan, Belarus, Georgia, Moldova and Ukraine, Jelochnik and Ivolga [45] (Russia and Serbia), Millns [46] (Armenia, Georgia and Moldova), Swinnen et al. [47] (Kazakhstan, Russia and Ukraine) and Wicki et al. [48] (Poland and Ukraine). Those studies were mainly based on the document analysis, descriptive statistics, comparison and synthesis methods applied. Few studies have used other research methods–for instance Hrybau et al. [49] used per capita intensity indices, the change rate index and a logistic function, while Timofti [50] incorporated the indicators of economic efficiency and synthetic indicator for (full) efficiency. Nevertheless there are not too many studies that would show the impact of the diversification of the production potential of agriculture on the diversification of productivity and thus on the level of development of the agricultural sector of the CC and the EPC analyzed together. Thus this paper analyses a current and actual topic—investigating the regional diversification of the EU candidate and Eastern Partnership countries.

In view of the above, the purpose of this paper is to determine how the diversification of the agricultural sector’s production potential translates into the diversification of the sector’s productivity and thus the achieved level of sector development of the CC and the EPC and then to characterize the groups of countries by the level of development and selected variables related to agriculture situation. This allows to identify the current status of agricultural sector in different countries (based on the latest available data), considering both aspects (production potential and productivity) combined together. This paper adds value to the existing literature mainly by providing an international approach to effectiveness, addressing both potential and productivity aspects. In this respect, the approach proposed in this paper differs from other analyses cited above. Therefore, an important advantage of this study is a synthetic approach to the development level of the agricultural sector issue, enabled by the use of a synthetic indicators which take production potential and productivity into account, and provide a basis for the typological classification of the CC and the EPC. This allowed to diagnose the areas which differ from each other in both aspects: the production potential and productivity.

The study of the diversification of the level of development of the agricultural sector using statistical methods is an important element in testing economic theories in the field of competitiveness research in the CC and EPC and it shows which countries may become competitive in the future in the EU market. Showing the importance of the diversification of the level of development of the agricultural sector in the CC and the EPC we can observe its impact on the future integration process of the CC and EPC. The results of the research are important from the point of view of the contribution to the economy of European integration, both in theoretical and empirical terms, but above all in the field of sectoral policy, which is the Common Agricultural Policy (CAP). A comprehensive approach to the subject of research and its multidirectional nature, as well as the obtained results will be important both for the agricultural policy of the studied countries, as well as for the CAP.

As far as the limitations of the study are concerned the author is aware that because of the approach used and of its international nature, this study fails to take into account a number of major aspects of the development level of agricultural sector, such as for instance the historical or the institutional country-level variation in the aspects considered. Extending analysis to examine the above mentioned issues will be the basis for further research. Nevertheless, the main goal of this approach was to emphasize the importance of diversifying of the production potential factors influencing on diversifying of achieved productivity and thanks to this achieved agricultural sector’s development level at the international level.

The rest of this article is organized as follows: Section 2 presents the data and research methods; Section 3 presents the research results and their discussion; and Section 4 presents a summary of the analysis.

## Materials and methods

The analyses were conducted for the candidate countries to the EU such as: Albania, Bosnia and Herzegovina, Montenegro, Macedonia, Serbia and Turkey, and the Eastern Partnership countries: Armenia, Azerbaijan, Belarus, Georgia, Moldova and Ukraine. In order to present the diversity of production potential and productivity in CC and EPC, the changes in the agricultural sectors of these countries in the years 2006–2017 were analysed in detail.

The lower time range adopted in the study resulted from the absence of reliable data for Serbia and Montenegro prior to 2006, since, before that time, the countries shared a single statistical office. The data employed in this study for comparative purposes was obtained from a single FASOTAT database, while in order to assess the changes in production potential and productivity, both the pattern and the non-pattern methods were implemented to calculate a synthetic measure of development, which is a function of aggregating partial information contained in individual variables.

The linear ordering is based on the necessity to distinguish between the output set of diagnostic variables of the three subsets. The first one contains stimulants, the second destimulants, while the third–dominants [51]. The features cannot be too heavily correlated with one another, as it results in a multitude of information repetitions entered into the system. In theory, the number of all variables may be substantial, however, in order for the analysis to be practically useful, their amount should not be too extensive [52]. The fundamental goal of the multidimensional comparative analysis (MCA) is to organize the set of units according to a specific criterion from "best" to "worst". A synthetic measure of development (SMD) is the tool required to carry out this procedure [51]. The synthetic measure of development can be determined by means of the pattern and non-pattern methods. The difference is that the construction of the first measure has a specific reference point, based on which the level of development will be determined [53]. The construction of the synthetic measure involves five stages:

Stage I–propositioning of a list of diagnostic features,
Stage II–normalization of diagnostic feature values,
Stage III–construction of a synthetic measure of development,
Stage IV–distinguishing the typological classes and the characterising of types,
Stage V–assessment of the production potential and productivity of agriculture.

In the first stage, simple features (partial variables) characterizing a given structure in a system of spatial units depending on the nature of the research are determined, most frequently based on the substantive premises. Therefore, the aggregate (synthetic) indicator is established on a set of features that directly determine the properties of units from the studied population [54]. Zeliaś [55] notes that too many features may disturb or even obstruct the prospect of effective unit classification. For the taxonomic analysis, it is preferable to select indicator variables. Remaining by the values in absolute terms may lead to falsified results [53]. In the set of variables (simple features) characterizing the examined units, elements with different directions of preferences may appear in relation to the properties of the considered partial structure. Among the simple traits that constitute a synthetic feature may appear elements called stimulants, destimulants and nominants. A simple feature is considered to be a stimulant when it positively correlates with a synthetic trait, i.e. a trait whose higher values are desirable, contrary to the low values, with regard to the considered synthetic property. The destimulant’s feature is negatively correlated with the synthetic trait, i.e. its lower values are desirable, while high values are considered adverse, with regard to the considered synthetic feature. At last, the nominant’s feature does not exhibit any significant correlation with the synthetic feature–in a certain range, it acts as a stimulant, and in other as a destimulant (it is possible to determine its optimal number) [53, 56, 57]. Establishing the nature of variables should be conducted on the basis of actual data characterising the phenomenon, i.e., supported by the non-stochastic (substantive) premises, although, in the absence of an appropriate theory, the expert evaluation method may be applied [56].

The selected simple features are then assigned with weighting factors: constant (individual) if equal importance of the traits is assumed, or differentiation if their contribution to the aggregate trait is to be diverse. The same applies to units. The determined feature values for individual statistical units are then compiled into a data matrix [56]:

Features

$$X=[\begin{array}{cccc} x_{11} & x_{12} & \ldots & x_{1K}\\ x_{21} & x_{11} & \ldots & x_{1K}\\ \ldots & \ldots & \ldots & \ldots \\ x_{N1} & x_{N2} & \ldots & x_{NK} \end{array}]\;\mathrm{Units}$$ (1)

where x_ik_ (*i* = 1, 2,…, N; *k* = 1, 2,…, K) represents the value of a simple feature no. *k* in a statistical unit no. *i*. This matrix constitutes an initial point in the construction of a synthetic feature.

The second stage involves a normalisation process, whose main purpose is to unify the character of the traits by transforming the destimulant and the nominant into stimulants (the postulate of uniform preference) and to reduce their value to comparability (the postulate of additivity), which consists in depriving them of their labels and unifying the orders of magnitude. The features adopted in the study were normalised by adopting a standardization formula. Conversion of the destimulant and nominant into the form of a stimulant:

- the nominants are transformed into stimulants according to the following formulas:

$$a)\;z_{ij}=\frac{x_{ij}}{nom\;\{x_{ij}\}},\;x_{ij}\leq nom\;\{x_{ij}\},\;nom\;\{x_{ij}\}\neq 0,$$ (2)

$$b)\;z_{ij}=\frac{nom\;\{x_{ij}\}}{x_{ij}},\;x_{ij}>nom\;\{x_{ij}\},\;x_{ij}\neq 0.$$ (3)

nom {x_ij_} –the nominal value of the feature no. *j*, considered as optimal or desirable, which can be determined based on:

–expert evaluations ($\underset{i}{\max}\;\{x_{ij}\}$, $\underset{i}{\min}\;\{x_{ij}\}$, nom {x_ij_}).

In the third stage, the non-pattern or pattern methods are used to construct a synthetic measure. The non-pattern method consists in averaging the values of the analyzed features. The average and standard deviations are calculated based on the obtained values, which allows for the division of the analyzed countries. Higher values indicate a greater degree of variation in production potential or productivity, while lower values demonstrate a reduced degree of variation.

The pattern method consists in calculating the Euclidean distance (*z*_*ij*_) from the model unit based on the normalised values of simple features, obtaining a synthetic Hellwig’s measure (*d*_*i*_) both for the production potential and for their productivity in the studied units–countries. Distance from the model unit is calculated for each country, most frequently using the Euclidean measure in the form of:

$$d_{i0}={\left[\sum_{j=1}^{m}{\left(z_{ij}-z_{0j}\right)}^{2}\right]}^{\frac{1}{2}},\;i=1,2,\ldots ,m$$ (4)

the synthetic measure is ultimately defined as:

$$s_{i}=1-\frac{d_{i0}}{d_{0}},\;i=1,2,\ldots ,m,$$ (5)

where:

$$d_{0}={\bar{d}}_{0}+2S(d_{0}),$$ (6)

whereas:

$$arithmetic\;mean\;of\;the\;feature\;{\bar{d}}_{0}=\frac{1}{n}\sum_{i=1}^{n}d_{i0};$$ (7)

$$standard\;deviation\;of\;the\;feature\;S(d_{0})={\left[\frac{1}{n}\sum_{i=1}^{n}{\left(d_{i0}-{\bar{d}}_{0}\right)}^{2}\right]}^{\frac{1}{2}}.$$ (8)

where *d*_*0j*_ is the normalized value of the feature no. j for the model unit, represented by the vector d = (*d*_*01*_, *d*_*02*_,…, *d*_*0m*_) [58]. Maximum values for each feature are regarded as components of the vector **z**_0_. As a result, a synthetic measure is obtained. Most frequently, this measure assumes values from the range (0,1), however, values outside this range are possible as well. Values which are higher and close to one indicate a significant level of the examined phenomenon, while values close to zero–its low level. A negative value of the measure may occur when the situation of a given country during the examined period is visibly poorer than the situation of other states [59].

In the fourth stage, the determined values of the synthetic measure are employed for the linear ordering of units and–on this basis–for distinguishing their typological classes, supported by the analysis of differences in the level of the measure values, calculated for adjacent units, which have been ordered according to decreasing measure values [54].

These methods indicate which of the countries exhibits a higher degree of production potential or productivity and therefore occupies the first place in the adopted classification, and which of them are placed on further positions, i.e. possess a lower level of potential or productivity, as well as provide information on the characteristics of this division.

Additionally, a linear regression model was developed to present the correlation between production potential and productivity in the countries studied.

## Results and discussion

The resources of the production factors or the stream of their inputs are fundamental elements of the agricultural production potential, which depends on their quantity, quality and mutual correlations [60]. The production potential of agriculture is defined as the totality of natural and artificial factors which can be implemented in the production of agricultural products. The components of the agricultural production potential considered to be the most relevant are: labour resources; natural environment–land resources used for agriculture, soil quality, climate, terrain, water conditions; tangible means of production–fixed and working assets; intangible components–knowledge, mastered technologies, organization, etc. [61].

The individual countries surveyed dispose of diverse resources of agricultural production means resulting from the occupied area and the achieved level of economic development [5]. Due to the location of the countries in different climate zones and the diverse level of economic development, agriculture is considered to be complementary in many fields within their borders. Individual countries may produce products that other states cannot, or should not, due to insignificant production effects [62], which to some extent will affect the rate and scale of development processes in individual CC and EPC, and thus changes in the diversity of their production potential, which influence the variety of productivity.

The experience of global agricultural development indicates that, with the transition from lower to higher development stages, i.e. the higher level of the produced GDP, the structure and correlations of the agricultural production factors, the structure of farms, as well as the level and efficiency of the inputs, for the most part initially adopt a constant growing tendency, and then the amount of applied inputs stagnates, while the intensive growth factors begin to dominate.

Major structural changes relate to ownership, technical and production, as well as organizational and institutional transformations. The policy of each of the CC and EPC examined is targeted at accelerating the positive trends which intensify the structural changes in agriculture and rural areas. The more relevant ones include rationalisation of inputs and increasing their efficiency, as these processes create an opportunity to improve the situation in the agricultural sector.

The agricultural production process comprises of three basic production factors: land, labour and capital. The number of resources owned and the ability to utilise them influence the productivity and efficiency of agriculture, as well as the prospect of agricultural development. These factors determine the chances of competitiveness in the agricultural sector [6].

The method of distinguishing developmental types, which essentially aims at the construction of a synthetic measure was used to assess the degree of utilization of the production potential [57]. The level of agricultural development indicates both qualitative and quantitative changes that occur in the agricultural sector during the studied period. Determining the degree of agricultural development is a complex phenomenon, as the change of one indicator results in the change of the others. Occasionally, difficulties occur with regard to the selection of identical indicators for selected research units, as well as the ability to obtain data for selected time periods. In order to assess the level of agricultural development and therefore the diversity of production potential in the analysed countries, the following indicators were used:

- share of people employed in agriculture,
- number of employees per 100 ha of agricultural land,
- share of the agricultural land in the country’s area,
- the agricultural land’s area per 1 inhabitant,
- share of the arable land in the agricultural land’s area.

The above-mentioned variables were used for calculations, including both the substantive and statistical assumptions. Having considered the assumption that variables cannot be too heavily correlated with one another, the highest value was obtained on the diagonal inverse of the diagonal matrix 2.9. The indicators were selected in a manner, in which their values were characterised by a variable variation, were not excessively correlated and became comparable. For this purpose, the variable–the share of people employed in agriculture–was removed from the analysis. The indicators were subsequently divided into stimulants and destimulants. A stimulant indicates a trait whose high values are desirable and low ones are not, while a destimulant involves the contrary–low values are preferable while high ones are not [54].

To determine the changes occurring in the diversification of the agricultural potential, two, previously mentioned methods–pattern and non-pattern–were used.

Table 1 presents the classification of the analyzed countries by the level of agricultural production potential, calculated according to both methods. The measure values for individual countries indicate a strong variation in their level of productive potential of agriculture. The application of the above methods allowed for the division the analyzed countries into four groups. In the non-pattern method, the values of the obtained measures indicate that in 2006 and 2017, Belarus and Ukraine comprised the first group with the highest agricultural production potential, while the average value of the analyzed index for these countries in 2017 amounted to around 0.89. Such a high level of agricultural production potential has been achieved, among others, due to a small number of employees per 100 ha of AL, which is the lowest among the analyzed countries.

**Table 1:** Classification of EU candidate and the Eastern partnership countries according to the level of agricultural production potential, using the pattern and non-pattern methods in 2006 and 2017.

Non-pattern method		Pattern method		Number of employees per 100 ha of agricultural land	Share of the agricultural land in the country’s area (%)	The agricultural land’s area per 1 inhabitant (ha)	Share of the arable land (%)
group	country	group	country				
2006							
I	0.767 Belarus	I	0.647 Belarus	5.8	43.2	2.2	61.8
	0.735 Montenegro		0.531 Ukraine	4.2	37.3	2.2	33.8
	0.733 Ukraine		0.508 Montenegro	9.9	68.4	1.3	78.6
II	0.632 Moldova	II	0.428 Macedonia	18.2	73.4	0.8	73.3
	0.568 Macedonia		0.397 Turkey	6.9	47.6	1.2	35.9
	0.567 Turkey		0.347 Moldova	12.6	51.6	1.1	56.8
III	0.532 Serbia	III	0.301 Serbia	19.2	40.0	1.0	73.4
	0.454 Azerbaijan		0.230 Azerbaijan	33.5	54.9	1.0	38.7
	0.424 Armenia		0.193 Albania	36.9	59.1	1.0	25.8
	0.422 Albania		0.180 Armenia	56.5	39.0	0.9	52.1
IV	0.379 Georgia	IV	0.132 Georgia	47.3	36.1	1.6	18.4
	0.315 Bosnia and Herzegovina		0.098 Bosnia and Herzegovina	9.5	17.0	0.8	18.1
2017							
I	0.933 Ukraine	I	0.857 Ukraine	7.5	71.0	0.9	78.8
	0.845 Belarus						
			0.696 Belarus	5.9	42.0	0.9	66.2
	0.759 Moldova			17.0	75.0	0.7	74.1
II	0.589 Serbia	II	0.544 Moldova	17.2	40.0	0.5	74.7
			0.411 Macedonia				
			0.411 Turkey				
III	0.563 Turkey	III	0.394 Serbia	15.4	50.0	0.5	53.6
	0.560 Macedonia		0.279 Azerbaijan	12.0	50.0	0.6	32.9
	0.491 Azerbaijan		0.272 Armenia	38.8	58.0	0.5	40.6
	0.488 Armenia		0.259 Bosnia and Herzegovina	28.6	59.0	0.6	26.6
	0.475 Bosnia and Herzegovina		0.243 Albania	9.5	20.0	0.7	19.7
	0.453 Albania		0.221 Montenegro	45.3	43.0	0.4	52.4
	0.423 Montenegro	IV	0.180 Georgia	8.6	17.0	0.4	29.8
	0.412 Georgia			32.7	37.0	0.7	17.6

Source: Own study based on data from FAOSTAT 2020 [63]: www.fao.org/faostat/en. The countries’ indicators are arranged according to the non-pattern method.

**Fig 1:**
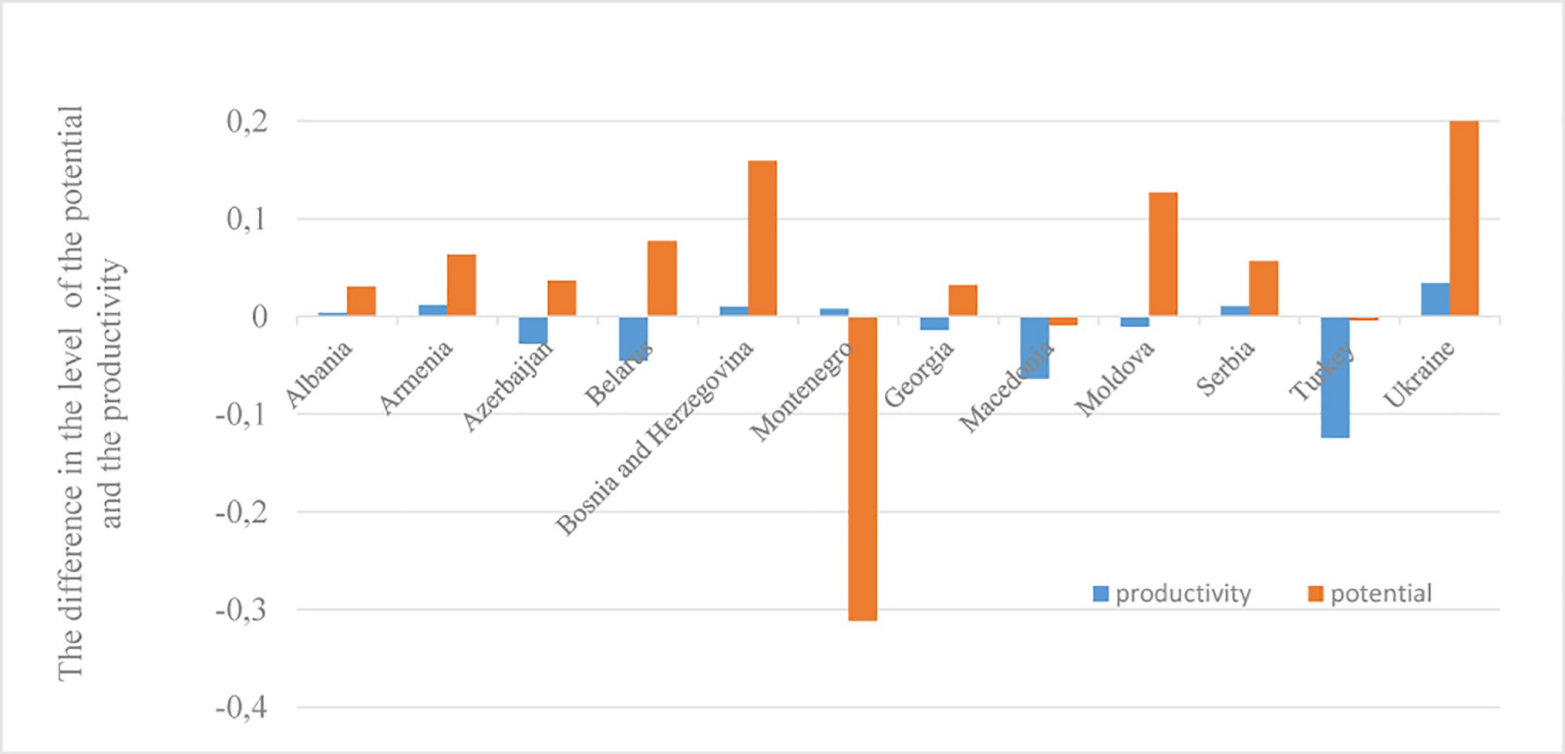
The difference in the synthetic measure of the agricultural potential and productivity level (calculated using the non-pattern method) for the studied countries in 2006 and 2017. Source: Own study based on Tables 1 and 2.

The high position of these countries also results from the largest area of AL per 1 inhabitant. In 2017, Moldova joined the group I, which recorded one of the most significant increases in the value of the analyzed index (by 0.127) in the compared periods (Fig 1). Such placement was a consequence of a decrease in employment per 100 ha of AL, an increase of both the share of the AL in the country’s area, as well as the share of the arable land in the AL’s area. However, the greatest increase in agricultural production potential occurred in Bosnia and Herzegovina (by 0.160), promoted from group IV (2006) to group III in 2017. The country recorded an increase in the share of AL in the country’s area, as well as the share of the arable land in the AL’s area. The country which noted an increase in the production potential of agriculture, ensuring the change of position in the classification from group III (in 2006) to group II (in 2017) was Serbia, in which employment per 100 ha of AL was reduced while the share of the arable land in the AL’s area increased. On the other hand, despite the fact that Georgia changed its position from IV (in 2006) to III (in 2017), the country exhibited the lowest agricultural production potential in both examined periods. The low level of the analyzed index resulted, among others, from the insignificant share of both AL in the country’s area (37%), as well as the arable land in the AL’s area (about 18%), and simultaneously a small AL’s area per inhabitant (0.7 ha).

Comparing 2006 and 2017, the most notable decrease in the level of agricultural production potential was observed in Montenegro, which in 2006 was placed in group I, next to Ukraine and Belarus, while in 2017 –only in group III. The decline of the analyzed measure in 2017 in Montenegro, resulted mainly from a two-fold increase in the number of employees per 100 ha and a nearly five-fold decrease in the AL’s area per inhabitant. Countries that observed an insignificant decline in the level of agricultural production potential, but which influenced their position in the compared periods (from group II in 2006 to group III in 2017) are Macedonia and Turkey. In the former, it resulted from an almost two-fold increase in the number of employees per 100 ha of AL and a decrease of AL per 1 inhabitant, in the share of arable land in the AL’s area. However, an increase in the number of employees per 100 ha, a decline in the share of AL in the country’s area and the arable land in the AL’s area, as well as a two-fold decrease in the AL’s area per inhabitant were recorded in Turkey during the studied period. The rest of the studied countries in the analysed periods remained within the same groups (II and III), which results from very insignificant changes in their agricultural production potential.

The findings obtained using the pattern method present a similar division of countries in terms of the level of agricultural production potential as the non-pattern method. This concludes, that the highest agricultural production potential in 2017 and 2006 occurred in Belarus and Ukraine. According to both the pattern and non-pattern methods, an increase in agricultural production potential was recorded in Bosnia and Herzegovina while its decrease in Montenegro. Moreover, the applied methods showed that Georgia is the least developed with regard to agriculture. In the rest of the countries, the level of production potential was comparable and they remained within the same groups in the studied periods.

Assessment of the level of the agricultural production potential is a complex process due to the numerous and diverse indicators characterising the agricultural situation in the countries concerned. The diversification of potential greatly influences the diversity of the obtained agricultural sector’s productivity as well. In order to determine the diversity of the agricultural sector’s productivity, synthetic measures of agricultural development were calculated for both 2006 and 2017 (Table 2). The analysis was originally conducted using the following indicators: the value of agricultural production per employee, per hectare of AL, as well as per farm. However, having considered the assumptions provided in the methodology, the variable production value per hectare of AL was removed due to its significant level of correlation. That is why the highest value on the diagonal inverse of the diagonal matrix amounted to 1.6. The analysis conducted with the use of both the pattern and non-pattern methods revealed that, although in 2006 and 2017, a certain fundamental regrouping of the examined countries’ ranking order in terms of agricultural productivity took place, three groups of countries emerged. In both examined periods, Belarus was placed in the first group, with a value of agricultural production per farm which differentiated it from other countries.

In 2017, according to the non-pattern method, with regard to agricultural productivity levels, Montenegro also ranked within the first group, as in that year the country exhibited a significant increase in both the volume of production per employee, as well as per farm. In group III, in 2017, compared to 2006, Bosnia and Herzegovina, as well as Serbia, advanced to higher positions, while other countries experienced slight changes. Comparing the values of the production per employee, it can be stated that in 2017, compared to 2006, the rates increased in the majority of analysed countries, with the exception of Azerbaijan, Georgia, Turkey and Macedonia. The highest labour productivity growth in the agricultural sector was recorded in Bosnia and Herzegovina (by 75%) and Ukraine (by 67%). Only five countries displayed an increase in the production per farm, where the largest took place in Serbia. In the future, the observed rise in the production capacity in these countries may result in the increased competitiveness of their agricultural sectors.

**Table 2:** Classification of EU candidate and the Eastern partnership countries according to level of the agricultural productivity, using the pattern and non-pattern methods in 2006 and 2017.

Non-pattern method		Pattern method			Production/employee	Production/farm
Group	country	Group	country		Euro	
2006						
I	1.000 Belarus	I	1.000 Belarus		16713.5	2055556.0
II	0.494 Montenegro	II	0.457 Montenegro		16471.8	6741.1
	0.372 Turkey		0.442 Turkey		12216.7	25411.7
	0.253 Ukraine		0.423 Ukraine		5222.2	398176.8
III	0.207 Macedonia	III	0.369 Macedonia		6861.3	5753.9
	0.168 Serbia		0.346 Serbia		5551.9	7168.6
	0.088 Armenia		0.294 Armenia		2898.5	6617.1
	0.087 Albania		0.294 Albania		2837.9	8941.3
	0.072 Moldova		0.283 Moldova		2385.0	2112.6
	0.065 Azerbaijan		0.278 Azerbaijan		2111.1	5404.2
	0.040 Bosnia and Herzegovina		0.259 Bosnia and Herzegovina		1311.1	1669.2
	0.035 Georgia		0.256 Georgia		1151.4	2386.3
2017						
I	0.955 Belarus	I		0.950 Belarus	21016.0	2607075.1
	0.502 Montenegro	II		0.451 Montenegro	23108.3	11028.8
II	0.287 Ukraine			0.440 Ukraine	8741.4	512407.2
	0.247 Turkey			0.384 Turkey	11228.1	22077.2
III	0.178 Serbia	III		0.345 Serbia	8104.8	16049.4
	0.143 Macedonia			0.323 Macedonia	6561.8	5184.5
	0.100 Armenia			0.294 Armenia	4577.7	6073.0
	0.091 Albania			0.288 Albania	4176.9	4078.5
	0.062 Moldova			0.267 Moldova	2835.0	1316.6
	0.050 Bosnia and Herzegovina			0.258 Bosnia and Herzegovina	2291.2	2651.5
	0.037 Azerbaijan			0.249 Azerbaijan	1688.1	2585.1
	0.021 Georgia			0.237 Georgia	976.8	1065.7

Note–countries were characterised according to the non-pattern method. Source: Own study based on data from FAOSTAT 2020 [63]: www.fao.org/faostat/en.

To assess the strength of the correlations between production potential and productivity of the agricultural sector of CC and EPC, a linear regression analysis was conducted. The synthetic measure of the production potential level for 2006 and 2017 respectively was assumed as the independent variable (x), while the synthetic measure of actual factor productivity for the studied periods–as the dependent variable (y). A number of conditions were adopted, assuming that the variation coefficient of each feature was higher than 0.1, the independent variables were less correlated with one other than with the dependent variable, the tolerance was higher than 0.1, the rectified R-square was as large as possible, and the inflation factor index of variance did not exceed 10.

Table 3 presents statistical characteristics of linear regression equations (y = ax + b), for the studied periods, as well as the values of regression of the coefficients B. These parameters, determine the theoretical level of productivity, while the regression equations assumed the following form: for 2006 y = 0.928 x + 0.083, and for 2017 y = 0.541 x + 0.159. While estimating the potential for 2006, it was proved that the regression coefficient of y on x amounted to 0.928, with a standard error of 0.229. The determined level of the Student’s significance test was t = 4.047 and the critical level of significance was set at 0.0023.

**Table 3:** Statistical characteristics of the regression equations of the correlations between production potential and agricultural productivity in the countries studied in 2006 and 2017.

**Year/2006**	**Summary of the linear function regression: Productivity y = f(potential (x))**							
	**R**^**2**^ **= 0.6209; Corrected R**^**2**^ **= 0.5830; F**_**(1,10)**_ **= 16.381 p < 0.05**							
	**Estimation standard error: 0.1321**							
	β	Standard error β	B	Standard error B	Partial correlation	Tolerance	t(10)	p
B_0_			0.0830	0.0853			0.9731	0.3534
Potential (x)	0.7880	0.1947	0.9278	0.2292	0.7880	1.000	4.0474	0.0023
**Year/2017**	**Summary of the linear function regression: Productivity (y) = f(potential (x))**							
	**R**^**2**^ **= 0.3321; Rectified R**^**2**^ **= 0.2653; F**_**(1,10)**_ **= 4.9727; p < 0.05**							
	**Estimation standard error: 0.1668**							
	β	Standard error β	B	Standard error B	Partial correlation	Tolerance	t(10)	p
B_0_			0.1594	0.1077			1.4801	0.1696
Potential (x)	0.5763	0.2584	0.5406	0.2424	0.5763	1.000	2.2299	0.0498

Source: Own calculations based on FAOSTAT 2020 [63]: www.fao.org/faostat/en.

The declared level of significance (0.05) was higher, therefore, this correlation might be considered statistically significant. The value of the so-called corrected coefficient of determination amounted to 0.583%, which indicated that 58% of the overall variable variation in factor productivity was explained by the model. In the model, the variable of production potential assumed a partial correlation coefficient of 0.79, which demonstrated that this variable alone explained the highest percentage of the productivity variance. While estimating the production potential for 2017, it was noted that the regression coefficient of y on x amounted to 0.541, with a standard error of 0.242. Student’s significance level t = 2.229 and significance level p < 0.050. The value of the corrected coefficient of determination R^2^ equaled 0.265%, which indicated that 27% of the total variable of the productivity factor variation was explained by the model. In the model, the variable of production potential assumed a partial correlation coefficient of 0.58. The linear regression analysis revealed that in 2017, this correlation was not statistically significant, as p = 0.169 which indicated a higher than the declared level (p<0.05). It is, therefore, confirmation of the changes in the analyzed measures of potential and productivity levels which occurred in 2017.

Furthermore, the study calculated the measure of the utilization of the agricultural production and economic potential degrees in individual countries, expressed as the ratio of the actual level of productivity to its theoretical level (Table 4). Values higher than 1 indicated that the actual level of productivity was greater than the theoretical, i.e. the production potential was properly utilised in a given country [64]. Table 4 indicates that the most significant increase in the analyzed index was recorded in 2017, in Montenegro. In the compared periods, the degree of utilization of the production potential increased in Belarus, Macedonia and Turkey as well.

**Table 4:** The quotient of the actual level of agricultural productivity per its theoretical level in the countries studied in 2006 and 2017.

	Albania	Armenia	Azerbaijan	Belarus	Bosnia and Herzegovina	Montenegro	Georgia	Macedonia	Moldova	Serbia	Turckey	Ukraine
2006	1.12	1.17	0.95	1.47	1.52	0.83	1.28	0.77	0.70	0.97	0.98	0.73
2017	1.00	0.95	0.81	1.77	0.87	1.61	0.93	0.84	0.59	0.94	0.99	0.71

Source: Own calculations.

The processes influencing changes in the agricultural production potential in individual countries led to a rise in its diversity among the CC and EPC, as evidenced by the slightly increasing value of the standard deviation (the standard deviation in 2006 amounted to 0.17, while in 2017–0.21). However, the diversity of the potential utilisation among the analyzed countries decreased (from 0.20 to 0.19). This took place regardless of the increase in production potential among the studied countries (with the exception of Montenegro and Macedonia) and a decrease in factor productivity (apart from Albania, Armenia, Bosnia and Herzegovina, Montenegro, Serbia and Ukraine) (Fig 1).

Reduction of the diversity in both the production potential and productivity of the agricultural sector in the studied countries is a long-term process which involves ensuring the possibility of the polarization and convergence occurrence. According to the theory of convergence, they reduce or even eliminate the existing disparities. In highly developed regions, the increase in profit with regards to the applied capital is smaller than in poor regions, while in the latter, the accumulation of capital rises, which results in the production growth at a much higher rate than in the wealthy territories. The market forces (not corrected by an appropriate intervention policy) incline towards the direction of polarization, not equalization of the socio-economic level of regions, which is particularly unfavourable for the CC and the EPC [65].

## Conclusions

1. The studied countries are characterized by widely ranged levels of agricultural production potential. However, the variation of the analyzed index in the concerned group is decreasing, as in 2007 it measured 4.9-fold while in 2017–4.8-fold. The countries with the highest level of agricultural development, regardless of the calculation method used, are Belarus and Ukraine. They are characterized by the lowest number of employees per 100 ha of AL among the surveyed countries, as well as the largest area of AL per inhabitant. The lowest level of the agricultural sector’s development was observed in Georgia. The country recorded a high level of employment per 100 ha of AL, as well as a low share of AL in the country’s area and arable land in the AL’s area.
2. The country which exhibited the highest level of agricultural productivity in the analyzed periods was Belarus, which is distinguished by the largest value of agricultural production per farm. In half of the countries surveyed, a decline in the value of the synthetic measure of agricultural productivity occurred within the analysed periods, while in the remaining half of them–a slight increase. The diversity among the analysed countries with regard to the level of agricultural productivity is comparable (about 4-fold) in both examined periods.
3. The conducted analyses indicate that CC and EPC’s agriculture is characterized by a significant spatial diversity, both in relation to the production potential, as well as the degree of its utilization, evidenced by the results of the comparison between the actually achieved level of factor productivity and its theoretical level. The transformations occurring within the structure of production, resources and the correlations between the production factors in the examined period have not significantly changed the diversity existing several years earlier. The spatial distribution of the agricultural production potential still indicates a significantly far better competitive ability of the countries of considerable size, where large state or cooperative farms previously existed. Therefore, the production potential of agriculture continues to be influenced by the shaped conditions and historical factors which fundamentally differentiate the studied countries, also reflected in the achieved productivity and their competitive position. The structural deficiencies of agriculture in smaller countries, belonging to both the former USSR and Yugoslavia, determine the low labour productivity of the sector, while relatively insignificant production intensity affects the poor land productivity. There are, however, certain modifications with regard to the extent to which the individual countries are utilising their production potential. The increasing potential in all analyzed states, with the exception of Montenegro and Macedonia, should be evaluated positively.
4. In conclusion, it can be stated that, within the analyzed group, two countries, dominant in terms of their production potential and agricultural productivity may be distinguished. These are Ukraine and Belarus. The collected data and calculated indicators prove that the countries possess a significant advantage over the others, as well as exhibit considerable potential to achieve competitive advantage in the market. Montenegro and Moldova display the potential to become a competitive country as well. Three states dominate the agricultural market: Belarus, Ukraine and Turkey. The country, in which a significant role is not attributed to the agricultural sector is Georgia. It results primarily from the climatic conditions (mountainous terrain), which largely limits agricultural production. In the remaining countries, the prospect of achieving competitiveness on the market of agri-food products is heavily restricted. It is based on the low level of countries’ development, the absence of investment in the agricultural sector, the lack of availability of foreign markets or poor level of productivity, which results from the structure of farms. The position of the agricultural sector was heavily influenced by the numerous economic transformations, particularly the agricultural reforms.
5. The level of agricultural development in the EU candidate and the Eastern Partnership countries is significantly lower compared to EU agriculture. The prospects of achieving competitiveness among these countries are often limited. The exceptions involve Turkey and Ukraine, which may obtain competitiveness due to large resources of production factors, mainly land, as well as Belarus, as a result of the favourable agrarian structure of farms.

## Abbreviations

AL: Agricultural land
CC: Candidate Countries
CAP: the Common Agricultural Policy
EPC: the Eastern Partnership countries
MCA: the multidimensional comparative analysis
SMD: the synthetic measure of development
